# Supplementary material for: PDE3A Is a Highly Expressed Therapy Target in Myxoid Liposarcoma
Source: Cancers (Basel). 2023 Nov 7;15(22):5308. doi: 10.3390/cancers15225308 (PMC10669966; doi:10.3390/cancers15225308)
Supplement: Supplementary file 1 [file cancers-15-05308-s001.zip › Supplementary Figures.pdf]

cancers

MDPI

Supplementary Figures

PDE3A is a highly expressed therapy target in myxoid liposarcoma

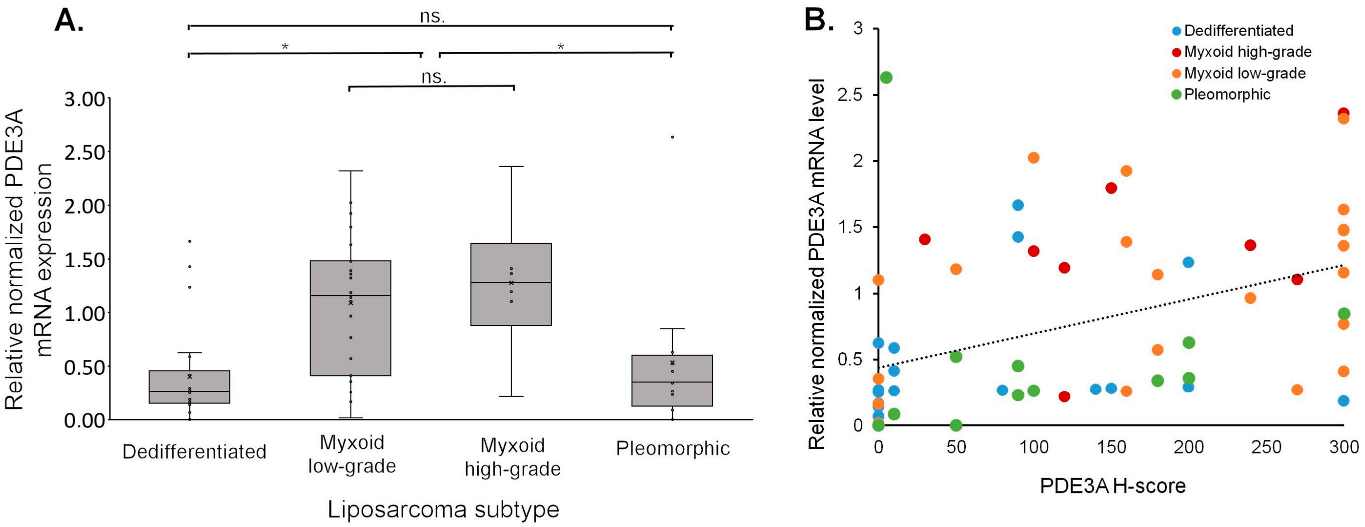

Figure S1. Validating PDE3A immunohistochemical staining in 63 LPS tissue results with RT-qPCR. (**a**) Strong PDE3A mRNA expression was observed in the myxoid subtype in comparison with other LPS subtypes. Data are presented as median relative to that of GIST882 cell line in FFPE (= 1). *p <0.01 (Kruskall-Wallis test). (**b**) PDE3A RT-qPCR and immunohistochemistry results showed a strong correlation (Spearman's ϱ = 0.423, $p < 0.001$). Each dot represents mRNA expression mean of (n = 3) biological replicates with the corresponding H-score value from immunohistochemical analysis of the sample. ns = not significant

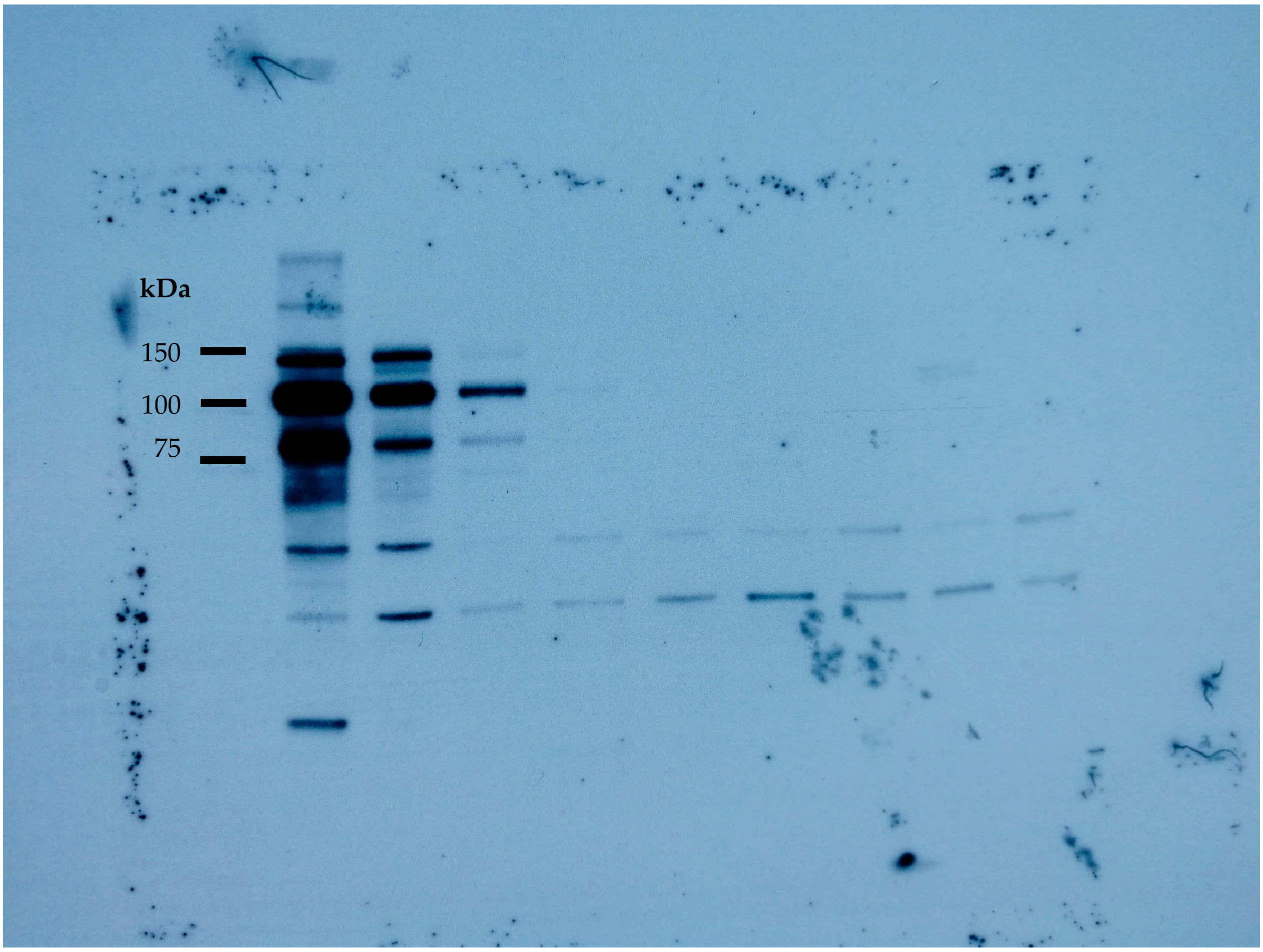

Figure S2. Unedited PDE3A western blot. 4 µg of protein/sample was loaded in wells in the following order: 1. GIST882, 2. SA4, 3. GOT3, 4. 93T449, 5.94T778, 6. LPS141, 7. MLS1765-92, 8. MLS402-91, 9. SW872

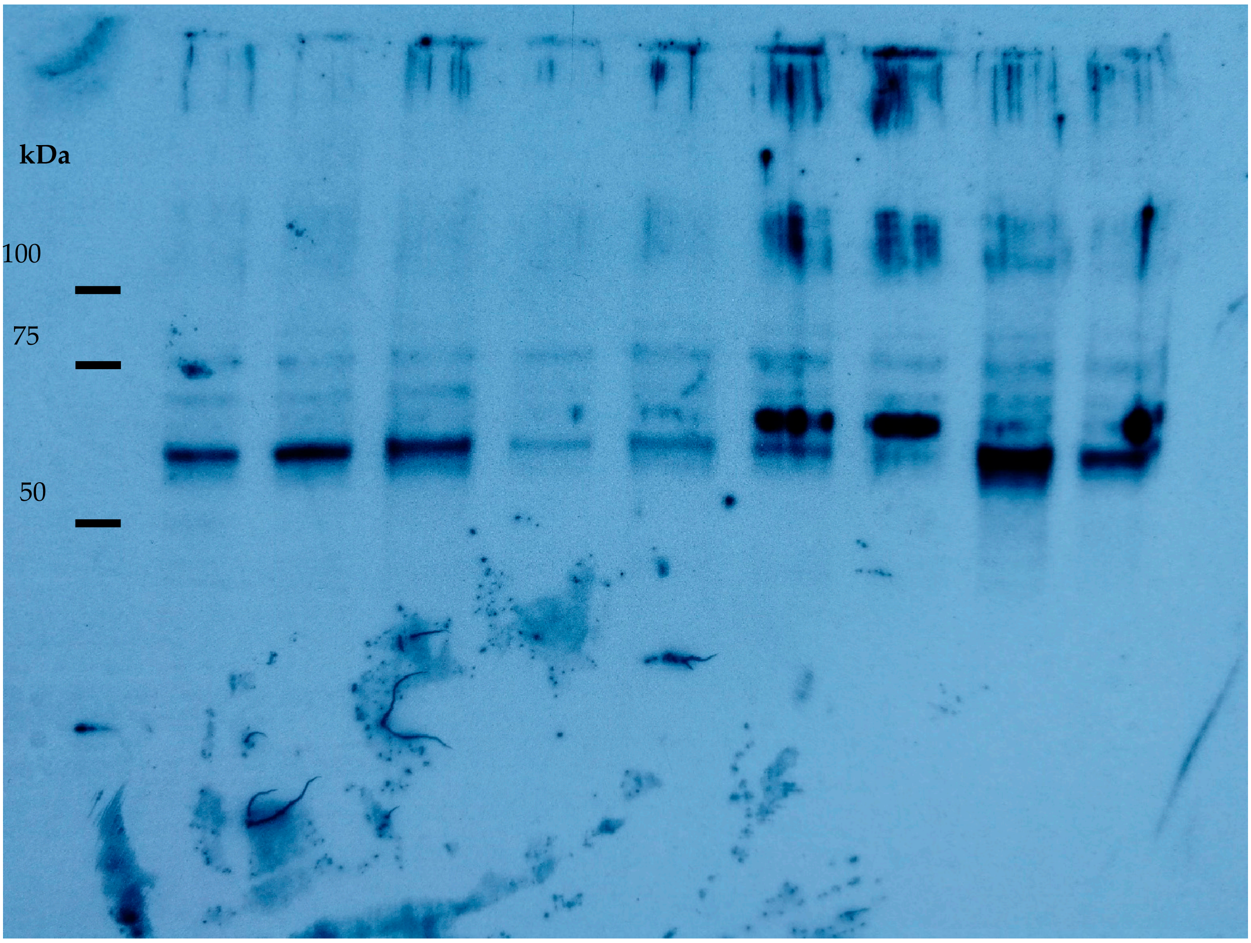

Figure S3. Unedited SLFN12 western blot. 4 µg of protein/sample was loaded in wells in the following order: 1. GIST882, 2. SA4, 3. GOT3, 4. 93T449, 5.94T778, 6. LPS141, 7. MLS1765-92, 8. MLS402-91, 9. SW872

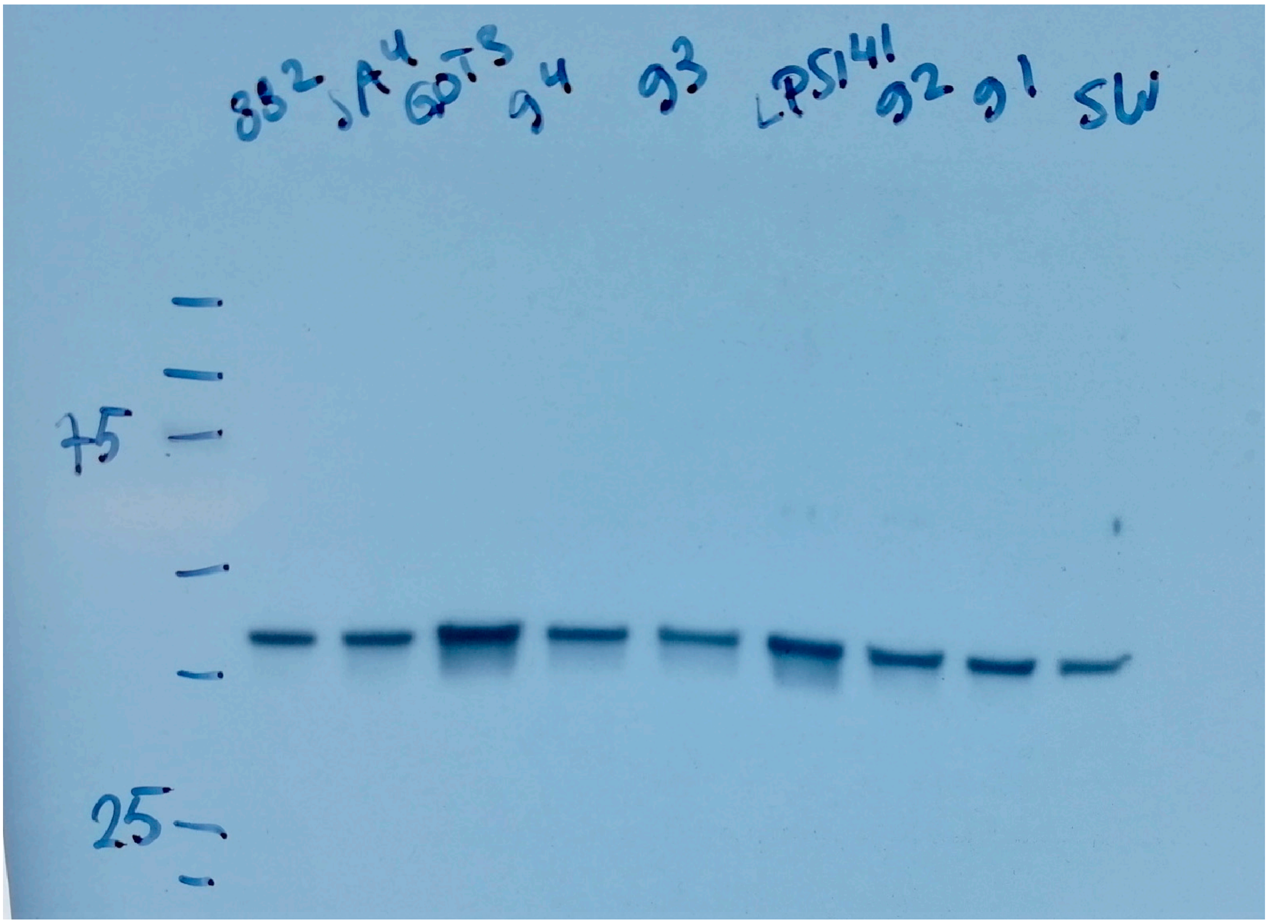

Figure S4. Unedited alpha actin western blot. 4 µg of protein/sample was loaded in wells in the following order: 1. GIST882, 2. SA4, 3. GOT3, 4. 93T449, 5.94T778, 6. LPS141, 7. MLS1765-92, 8. MLS402-91, 9. SW872
